# Histone H4 directly stimulates neutrophil activation through membrane permeabilization

**DOI:** 10.1002/JLB.3A0620-342R

**Published:** 2020-08-17

**Authors:** I‐Ni Hsieh, Xavier Deluna, Mitchell R. White, Kevan L. Hartshorn

**Affiliations:** ^1^ Department of Medicine Section of Hematology Oncology Boston University School of Medicine Boston Massachusetts USA

**Keywords:** respiratory burst, degranulation, calcium influx, monocyte, cytokine

## Abstract

Extracellular histones have been implicated as a cause of tissue inflammatory injury in a variety of disorders including sepsis, lung, and liver diseases. However, little is known about their interactions with neutrophils and how this might contribute to injury. Here, it is shown that histone H4 acts as neutrophil activator by inducing hydrogen peroxide production, degranulation, cell adhesion, and IL‐8 generation. Histone H4 caused permeabilization of the neutrophil membrane (a phenomenon described in other cell types) leading to accelerated cell death. H4 caused sustained rise in neutrophil intracellular calcium that is necessary for respiratory burst activation and degranulation. Convincing evidence was not found for TLRs or ATP receptors in H4 mediated activation. However, pertussis toxin and wortmannin (inhibitors of G protein and PI3K) inhibited H4‐induced hydrogen peroxide production and degranulation. These studies suggest that release of histone H4 in sites of infection or inflammation may potentiate neutrophil activation and promote additional inflammatory responses. These studies may provide a better basis for developing novel therapeutic strategies to block neutrophil extracellular trap (NET) and H4‐related pathology in sepsis and various forms of lung injury including that induced by viruses like influenza or SAR‐CoV2.

## Graphical Abstract

Histone H4 is a strong mediator of neutrophil activation, which could account in part for its profound inflammatory effects in vivo.

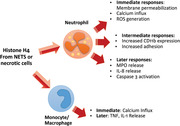

## 1 INTRODUCTION

Histones are positively charged, highly conserved proteins that are present in all eukaryotic cells. They are imported into the nucleus after synthesis in the cytoplasm, and they play essential role in organizing and stabilizing DNA by binding to it and forming the nucleosome, the basic unit of chromosome. The nucleosome consists of a 146‐bp DNA wound around a histone octamer, which is composed of 2 copies of core histones H2A, H2B, H3, and H4. H1 serves as the linker histone to connect nucleosomes into higher order structures. The N‐terminal tails of core histones have basic regions that provide sites for posttranscriptional modifications (PTMs), such as acetylation, methylation, or phosphorylation, which help in regulating chromatin structure and gene expression. In addition to the intracellular functions, histones have been found to have other functions in the extracellular space. The antimicrobial activity of extracellular histones was first discovered in 1942, since then their antimicrobial activity against bacteria, viruses, fungi, and parasites has been reported in various species.^1^, ^2^ We demonstrated that arginine rich histones (H3 and H4) are able to neutralize influenza virus in vitro.^3^

However, many studies now indicate that extracellular histones are predominantly harmful in vivo through profoundly inducing inflammation and activating coagulation. Histones have been shown to contribute to the pathophysiology of sepsis and various forms of lung injury (including influenza).^4^, ^5^, ^6^, ^7^, ^8^, ^9^, ^10^ In a sepsis model, histone H3 and H4 were found important factors contributing tissue injury and animal death, which were attenuated by administration of antibodies to histones.^11^ Another group also demonstrated that neutralization of histone H4 with IgG Ab ameliorated the severity of acute lung injury.^9^ Histones can be released from damaged or dying cells or through neutrophil extracellular trap (NET) formation in the case of neutrophils. The field of NET biology is rapidly evolving and it now appears that different pathways can lead to NET formation and that NET formation does not always result in neutrophil death. In addition, histones associated with NETs are not always citrullinated as originally thought.^12^ In any case, histone H4 is a major component of NETs and this appears to be one important source of extracellular histones. Extracellular histones have been considered as one group of the damage‐associated molecular patterns (DAMPs) that could trigger pro‐inflammatory signaling pathways upon binding to pattern recognition receptors (PRRs).^13^ Histones bind to platelets and induce profound thrombocytopenia in mice.^14^, ^15^ They enhance the procoagulant potential of human platelets and drive thrombin generation.^16^ Of great interest, in addition to the role of histones and NETs in severe influenza infection,^4^ recent evidence shows a role for neutrophil influx and NETs in COVID‐19 pathophysiology,^17^, ^18^ which includes profound inflammation and hypercoagulability. Hence, understanding mechanisms of action of histones in inflammation is a pressing goal.

The mechanism through which histones induce cytokine production and excessive coagulation is not fully settled. There are a number of articles indicating that binding to TLRs is a key event in cell activation by histones. For instance, Westman et al. showed that histone H4 binds directly to TLR4/MD‐2 complex on monocytes leading to the release of CXCL9 and CXCL10, which contributes to the recruitment of leukocytes to the site of infection.^13^ TLR2 and TLR4 were reported to mediate histone‐induced fatal liver injury in a mouse model^19^ and the ability of histones to activate platelets and induce thrombin generation was found to be mediated by platelet TLR2 and TLR4.^16^, ^20^ In a liver ischemia reperfusion injury model, extracellular histones were found to activate NLRP3 inflammasome resulting in caspase‐1 and IL‐1β secretion through acting on TLR9.^7^

However, other studies have raised a possible alternative mechanism for the pro‐inflammatory and hypercoagulability effects of histones. Histones have been shown to damage endothelial cells through cell permeabilization and calcium influx.^21^, ^22^ More recently, an elegant study showed that histone H4 causes chronic inflammation in a mouse model of atherosclerosis through causing lytic cell death of smooth muscle cells.^23^ This was mediated by insertion of histone H4 into the cell membrane and creation of membrane pores accompanied by calcium influx. In this study, a vicious cycle was demonstrated in which damaged smooth muscle cells in plaques recruited more neutrophils that released more histones as part of NETs. These studies suggest that histone H4 can activate inflammation through direct action on cell membranes and not necessarily through binding to known cell membrane receptors.

In this study, we demonstrate that histone H4 directly activates human neutrophils through a mechanism similar to that found by Silvestre‐Roig et al. for smooth muscle cells.^23^ We focused on predominantly on human histone H4 in part because we found, as did Abrams et al.,^21^ that other purified histone preparations available commercially contained endotoxin. In addition, histone H4 has been implicated in several studies of histone induced inflammation.^9^, ^23^ We characterize the different aspects of neutrophil function stimulated by histone H4 and found a key role of neutrophil intracellular calcium elevation in activation.

## 2 MATERIALS AND METHODS

### 2.1 Ethics statement

Blood collection for isolation of neutrophils and monocytes was done with informed consent as approved by the Institutional Review Board of Boston University School of Medicine. The Institutional Review Board specifically approved this study and also approved the consent form for the study. The blood donors were healthy volunteers and they all signed the written consent form prior to each donation.

### 2.2 Histone and other protein preparations

Recombinant histone H4 was purchased from New England Biolabs (Ipswich, MA, USA). Recombinant histone H2a, H2b, and H3 were purchased from Sigma–Aldrich (St. Louis, MO, USA). Endotoxin levels in histones was monitored by ToxinSensor chromogenic LAL endotoxin assay kit from Genscript (Piscataway, NJ, USA). PMA, fMLP, DPI, wortmannin, pertussis toxin (PT), and KN62 were purchased from Sigma–Aldrich (St. Louis, MO, USA). TLR2 and TLR4 Abs were purchased from Invitrogen (San Diego, CA, USA). LDH cytotoxicity detection kit was purchased from Takara (Kusatsu, Shiga Prefecture, Japan). Human IL‐1β ELISA kit was purchased from Peprotech (Rocky Hill, NJ, USA). TNF‐α ELISA kit was purchased from Thermo Fisher Scientific (Waltham, MA, USA). IL‐8 ELISA kit was purchased from BD Biosciences (Billerica, MA, USA). Propidium iodide (PI) was purchased from Abcam (Cambridge, United Kingdom).

### 2.3 Human neutrophil and monocyte/Mϕ preparation

Neutrophils from healthy volunteers were isolated to >95% purity by using dextran precipitation, followed by Ficoll‐Paque gradient separation for the separation of mononuclear cells (layering above the Ficoll‐Paque) and neutrophils (below the Ficoll‐Paque). The neutrophils were purified further by hypotonic lysis to eliminate any contaminating erythrocytes, as previously described.^23^ Cell viability was determined to be >98% by trypan blue staining. The isolated neutrophils were resuspended at the appropriate concentrations in control buffer (PBS) containing CaCl_2_ or MgCl_2_ (PBS++) and used within 2 h. For the experiments testing the requirement of extracellular calcium, PBS without CaCl_2_ or MgCl_2_ (PBS wo) was used. For the experiments testing the requirement of intracellular calcium, neutrophils were pretreated with BAPTA‐AM (20 μM) for 10 min. For mechanistic studies, neutrophils were pretreated with or without pertussis toxin (PT) (500 μg/ml) for 2 h, or wortmannin (1 μM) for 10 min, or TLR2 Ab (5 μg/ml) for 10 min, or TLR4 Ab (5 μg/ml) for 10 min, or DPI (20 μM) for 1 h, or KN62 (10 μM) for 1 h. DMSO was used as vehicle control for KN62 treated groups. Monocytes were isolated from the PBMC preparations by negative selection using magnetic beads and a Miltenyi monocyte isolation kit (catalogue number 130‐091‐153). Monocyte‐derived Mϕs were prepared by culturing adherent human monocytes in RPMI medium with 10% autologous serum (not heat inactivated) and 5 ng/ml of GM‐CSF. The media was changed every 3 days and assays were performed when the cells took on Mϕ appearance (∼10 days). THP‐1 cells were purchased from ATCC.

### 2.4 Measurement of neutrophil H_2_O_2_ production

H_2_O_2_ production was measured by assessing reduction in scopoletin fluorescence as previously described.^24^, ^25^ In brief, neutrophils were added to a mixture of scopoletin, sodium azide, and horseradish peroxidase, which were previously shown to maximize detection of H_2_O_2_. Results of this assay were previously correlated with results obtained by oxygen consumption, chemiluminescence, and other assays.^24^, ^25^, ^26^, ^27^ Measurements were made using a POLARstar OPTIMA fluorescent plate reader (BMG Labtech, Durham NC).

### 2.5 Measurement of neutrophil adhesion and integrin expression

The 96‐well plates were coated with gelatin (0.2% solution), human fibronectin (10 μg/ml), or left uncoated (plastic) for 1 h, followed by washing with PBS 3 times. Neutrophils were pre‐incubated with or without histone H4 for 20 min at 37°C, followed by washing with PBS 3 times. Neutrophils were then allowed to adhere to the plates for 20 min at room temperature. After washing with PBS, adherent cells were collected and solubilized by adding 200 μl of a 0.5% hexadecyltrimethylammonium bromide (Sigma) solution in 50 mM potassium phosphate buffer (pH 6.0). Intracellular myeloperoxidase (MPO) was measured with 3,3′,5,5′‐tetramethyl benzidine (TMB) as the substrate, and the reaction was stopped by adding 1 N hydrochloric acid (Sigma). The optical density (OD) of the sample was read at 450 nm wavelength with POLARstar OPTIMA plate reader (BMG Labtech, Durham, NC, USA).

For measurement of CD11b, neutrophils were treated with indicated concentrations of histone H4 for 20 min, followed by washing 3 times with PBS. Increase in expression of the neutrophil integrin CD11b was measured with FITC‐labeled anti‐CD11b Ab by flow cytometry.

### 2.6 Assessment of cytokine production

Neutrophils were incubated with or without histone H4 for 6 hours in a CO_2_ incubator in RPMI with 10% heat inactivated autologous serum. After which the supernatant was collected and assayed for IL‐8 using a commercially available ELISA kit (BD Biosciences, San Diego, CA) according to the manufacturer's instructions. Cells without any stimulus were used as negative controls in the experiment.

For measurement of TNF‐α or IL‐β in THP‐1 cells, human monocytes or monocyte‐derived Mϕs, cells were incubated with or without histone H4 for 24 h in a CO_2_ incubator in RPMI with 10% heat inactivated autologous serum. After which the supernatant was collected and assayed for TNF‐α or IL‐β using commercially available ELISA kits (BD Biosciences, San Diego, CA) according to the manufacturer's instructions. Cells without any stimulus were used as negative controls in the experiment.

### 2.7 Measurement of the release of MPO

Human neutrophils were treated with indicated proteins for 2 h. Samples were then centrifuged for 5 min at 400 × *g*, and supernatants were collected. Exocytosis of myeloperoxidase (MPO) was measured with 3,3′,5,5′‐tetramethyl benzidine (TMB) as the substrate, and the reaction was stopped by adding 1 N hydrochloric acid (Sigma). The optical density (OD) of the sample was read at 450 nm wavelength with POLARstar OPTIMA plate reader (BMG Labtech, Durham, NC, USA)

### 2.8 Measurement of neutrophil caspase 3 activity

Human neutrophils were treated with indicated proteins for 45 min, 2 h, or 5 h as indicated. Samples were then centrifuged for 5 min at 400 × *g*, and pellets were collected. Collected cells were then washed with PBS and lysed with lysis buffer. Caspase 3 activity of each sample was measured with EnzChek^®^ Caspase‐3 Assay Kit (Invitrogen™ Molecular Probes™). Ac‐DEVD‐CHO inhibitor was provided in the kit to ensure the observed signal is due to the activity of caspase‐3‐like proteases.

### 2.9 Measurement of intracellular calcium responses of neutrophils

Neutrophils were preloaded with Fura‐2AM for 30 min at 37°C. After washing, preloaded neutrophils at 2.5 × 10^6^ cells/ml were added to the 96‐well black plates (100 μl/well). PBS control, fMLP, or histone H4 was added at Time 135. Measurements were made using a POLARstar OPTIMA fluorescent plate reader (BMG Labtech, Durham, NC, USA).

### 2.10 Lactate dehydrogenase assay

The LDH assay was performed on neutrophils treated with or without histone H4 for 20 min. The assay was performed according to the manufacturer's instructions. In brief, the assay includes high control (LDH release in the cells by the addition of Triton X‐100) and low controls (spontaneous LDH release from untreated normal cells) and is an ELISA. The percent of LDH release is obtained from OD values by the formula: (OD490_sample_ – OD490_low control_) ÷ (OD490_high control_ – OD490_low control_) × 100.

### 2.11 PI staining

Neutrophils were treated with histone H4 or Triton X‐100 for 20 min, followed by washing 3 times with PBS. Samples were then incubated with 20 μg/ml PI for 5 min at room temperature in the dark, and the uptake of PI by neutrophils was measured by flow cytometry in FL3 channel.

### 2.12 Statistics

Statistical comparisons were made using Student's paired, 2‐tailed *t*‐test or ANOVA with post hoc test (Tukey's). ANOVA was used for multiple comparisons to a single control.

## 3 RESULTS

### 3.1 Effects of histone H4 on neutrophil functional responses

#### 3.1.1 Histones H4 stimulates neutrophil H_2_O_2_ production in a calcium dependent manner

**FIGURE 1:**
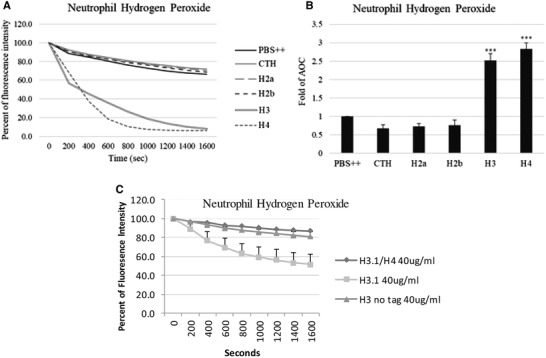
**Effects of extracellular histones on hydrogen peroxide production in human neutrophils**. Hydrogen peroxide production by neutrophils in response to 20 μg/ml histones was measured by assessing the reduction in scopoletin fluorescence. Fold changes of area over the curve (AOC) are shown respectively. CTH: calf thymus histones. *N* = 5. Panel (**A**) shows the mean time curves of the responses and panel (**B**) shows fold changes in area over the curve for these responses. Results are presented as mean ± sem (*P ≤ 0.05, **P ≤ 0.01, ***P ≤ 0.001). Panel (**C**) shows results for the complex of H3.1 and H4 and the N‐terminal fragment of H3.1 (H3 no tag) the responses were not increased compared to PBS alone. For H3.1, the responses were significantly greater than PBS alone (*P* < 0.01). Results are mean ± sem of 4 experiments with separate neutrophil donors.

Neutrophils activate antimicrobial killing programs when encountering infectious organisms. To kill and digest the microorganisms, neutrophils release the contents of intracellular granules into phagosomes and generate reactive oxygen species (ROS) through the activation of NADPH oxidase.^16^ We evaluated the ability of histones to induce neutrophil H_2_O_2_ production, and we found that histone H3 and H4 stimulated a significantly greater amount of H_2_O_2_ compared to histone H2a and H2b (Fig. 1A and B). We also tested the effects of a complex of H3 and H4 or a truncated form of H3 in comparison to full length H3 in this assay (Fig. 1C). Of note, neither the shortened form of H3 nor the complex of H3 and H4 induced H2O2 production. We tested for presence of endotoxin in the various preparations and found that H3, H2a, and H2b contained significant amounts of endotoxin (from 0.42 to 0.46 EU/ml) but H4 did not (<0.015 EU/ml). In parallel assays we did not find that this level of endotoxin induced neutrophil respiratory burst on its own; however, for subsequent experiments we focused our experiments on H4.

**FIGURE 2:**
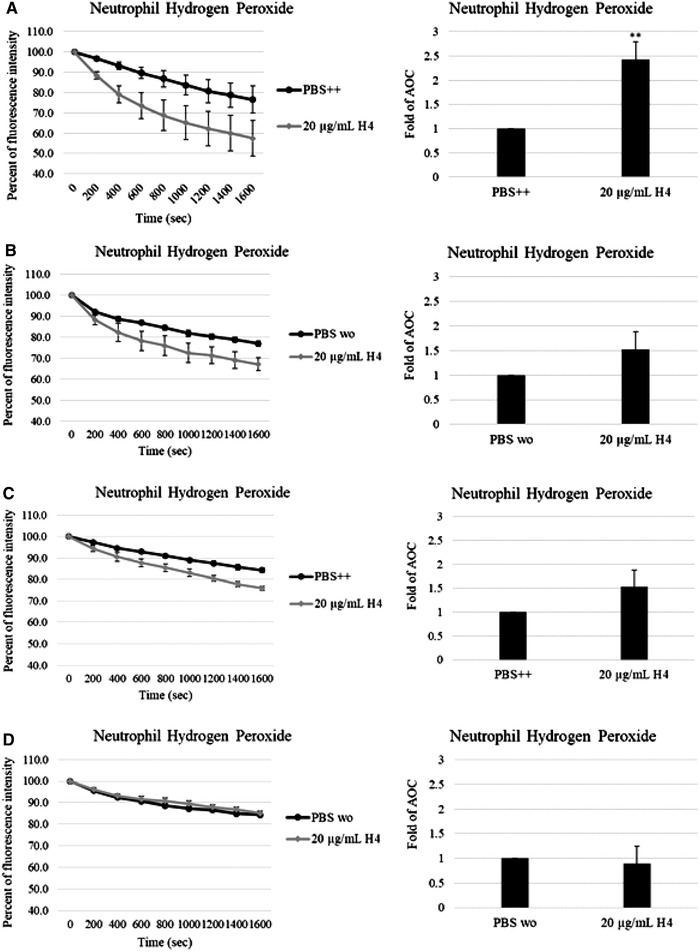
**Histone H4 induced hydrogen peroxide production in human neutrophils is calcium dependent**. Hydrogen peroxide production was measured by assessing the reduction in scopoletin fluorescence. Human neutrophils were treated without (**A** and **B**) or with 20 μM BAPTA‐AM for 10 min before the experiments (**C** and **D**), samples were made up with PBS++ (**A** and **C**) or PBS without calcium (**B** and **D**). Fold changes of area over the curve (AOC) are shown respectively. *N* = 5. Results are presented as mean ± sem (**P ≤ 0.01)

To identify metabolic events preceding this respiratory burst, we then evaluated the effects of restricting the availability of extracellular and/or intracellular Ca^2+^ sources. Incubation in Ca^2+^‐free buffer resulted in a 65% decrease in H_2_O_2_ production induced by histone H4 compared to control groups (Figs. 2A and B). We next examined the contribution of intracellular Ca^2+^ stores to histone H4‐induced H_2_O_2_ production. Neutrophils were pre‐incubated with BAPTA‐AM, an intracellular Ca^2+^ chelator, for 10 min at 37°C before the assay. The depletion of intracellular Ca^2+^ also led to ∼65% decrease in H_2_O_2_ production induced by histone H4 (Fig. 2C). When we pre‐incubated neutrophils with BAPTA‐AM and used Ca^2+^‐free buffer for the experiments, histone H4 caused no H_2_O_2_ production (Fig. 2D). These results indicate that neutrophil H_2_O_2_ production induced by histone H4 is strongly dependent on both extracellular and intracellular Ca^2+^.

#### 3.1.2 Histone H4 induced H_2_O_2_ generation is adhesion dependent and H4 potentiates neutrophil integrin expression and adhesion

**FIGURE 3:**
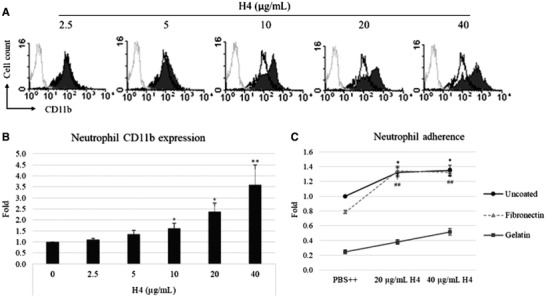
**Effect of histone H4 on human neutrophil adhesive responses**. (**A**) Neutrophils were treated with indicated concentrations of histone H4 for 20 min and the expression of integrin CD11b was measured by flow cytometry. Control cells that were not incubated with anti‐CD11b Ab are shown in each histogram overlay (gray peaks). Cells treated with control buffer PBS++ are shown in each histogram overlay (black peaks). Quantification of fluorescence intensity from flow cytometry is shown in (**B**). (**C**) Neutrophils were pretreated with indicated concentrations of histone H4 for 30 mins at 37°C followed by washing and adding neutrophils to 96‐well plates coated with 0.2% gelatin or 10 μg/ml fibronectin. Neutrophils were allowed to adhere for 20 min. Adhesion was measured by measuring myeloperoxidase (MPO) activity. Results shown in panels (**B**) and (**C**) are mean ± sem; *n* = 5 (*P ≤ 0.05, **P ≤ 0.01 compared to PBS control)

While histone H4 stimulated neutrophil H_2_O_2_ production in uncoated 96‐well plates, we found that it was unable to stimulate neutrophil H_2_O_2_ production when the plates were coated with 0.1% gelatin (data not shown). We previously found that gelatin coating could limit neutrophil adhesion to plates (data not shown) and thus this H_2_O_2_ production induced by histone H4 may be cell adhesion‐dependent. Mϕ‐1 Ag (Mac‐1) (CD11b/CD18), an integrin family member, is expressed by neutrophils and monocytes. The expression of Mac‐1 on the neutrophil surface may increase by rapid delivery of stores within neutrophil secondary granules in response to inflammatory stimuli. Mac‐1 has also been found to mediate adherence‐dependent H_2_O_2_ production in human neutrophils, so we next evaluated the effects of histone H4 on CD11b expression and neutrophil adherence. As shown in Figs. 3A and B, pretreatment of neutrophils with histone H4 for 20 min markedly increased CD11b expression on neutrophil surfaces in a concentration dependent manner.

To test the effects of histone H4 on neutrophil adhesion, we tested adhesion to plates coated with or without gelatin or fibronectin. While gelatin reduces neutrophil adherence, fibronectin is one of the Mac‐1 ligands. Unstimulated neutrophils were shown to bind the most to the uncoated (plastic) plates, and by comparison only 20% as many cells adhered to gelatin‐coated plates. Adhesion of histone H4‐treated neutrophils to all these plates was significantly increased, and the binding of neutrophils to fibronectin‐coated plates increased ∼62.5% compared to unstimulated neutrophils (Fig. 3C). The histone H4 induced increase in neutrophil CD11b expression likely accounts for increased adhesion to fibronectin‐coated surfaces.

#### 3.1.3 Histone H4 increases neutrophil degranulation and IL‐8 release

**FIGURE 4:**
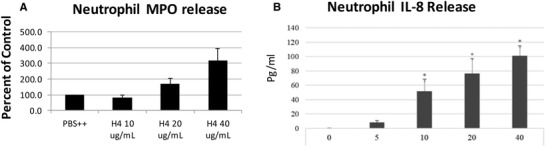
**Histone H4 induces release of myeloperoxidase and IL‐8 from human neutrophils**. (**A**) Neutrophils were treated with different concentrations of histone H4 for 2 h and then degranulation was assessed by measuring myeloperoxidase (MPO) release into the cell supernatant as compared to cells treated with buffer alone for the same amount of time. (**B**) MPO release was greatly reduced in buffer lacking calcium and magnesium compared to control buffer. (**C**) Neutrophils were treated with different concentrations of histone H4 for 6 h, and IL‐8 released in the supernatants was measured by commercially available ELISA kit. *N* = 5. Results are presented as mean ± sem (*P ≤ 0.05, **P ≤ 0.01, ***P ≤ 0.001)

A short incubation with histone H4 led to strong increase in CD11b on neutrophil surface, most likely due to recruitment of CD11b from pre‐formed stores in specific granules. We also tested effects of more delayed effects of histone H4 exposure on neutrophils. Primary or azurophilic neutrophil granules contain antimicrobial peptides and enzymes necessary for killing invading microorganisms. Excessive neutrophil degranulation, however, has also been considered responsible for various inflammatory disorders. Myeloperoxidase (MPO) is a well‐established bacterial killing enzyme stored in neutrophil primary granules, which can be released on stimulation. As shown in Fig. 4A, histone H4 significantly induced neutrophil release of MPO as compared to control buffer alone after 2 h incubation. IL‐8 (or CXCL8) is a key neutrophil chemotactic mediator playing critical roles in recruiting neutrophils to sites of infections, and can amplify an initial inflammatory response. We found that histone H4 strongly induced neutrophil IL‐8 release within 6 h (Fig. 4B).

### 3.2 Mechanisms of neutrophil activation by histone H4

#### 3.2.1 Histone H4 causes sustained elevation of neutrophil intracellular calcium

**FIGURE 5:**
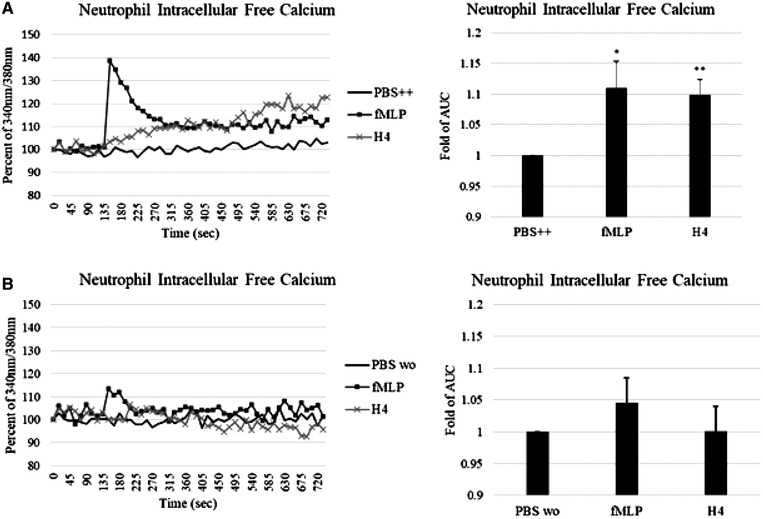
**Histone H4 causes influx of calcium from extracellular medium**. Intracellular free calcium changes on exposure to 40 μg/ml histone H4 in the presence (**A**) or absence (**B**) of extracellular Ca^2+^ were measured by detecting the fluorescence of Fura‐2 AM loaded neutrophils. fMLP (positive control) or histone H4 was added to the cells at 135 s. Fold changes of area under the curve (AUC) are shown. Results are mean ± sem for 4 experiments. Neutrophil depolarization was significant (*P* < 0.01) for H4 as compared to cells maintained in PBS alone

We expected that the H_2_O_2_ production induced by histone H4 was due to calcium flux reaction since store‐operated calcium entry (SOCE) has been considered critical for NADPH oxidase activation. fMLP, a neutrophil chemotactic factor released from bacteria, is a well‐established positive control as it causes a rapid increase in neutrophil intracellular calcium level that then returns to baseline in 3 min. Interestingly, histone H4 did not cause a typical calcium flux like fMLP, but rather led to a slower, progressive increase in neutrophil intracellular calcium level that never returned to baseline (Fig. 5A), suggesting that SOCE mechanism was not involved in histone H4‐induced calcium flux. To test the source of calcium contributing to the calcium flux, we measured the effects of extracellular calcium chelation on the responses using calcium‐free PBS buffer with added EGTA. Extracellular calcium chelation did not totally block fMLP‐induced calcium flux but it totally blocked histone H4‐induced responses, indicating that calcium flux caused by histone H4 was dependent on extracellular calcium (Fig. 5B).

**FIGURE 6:**
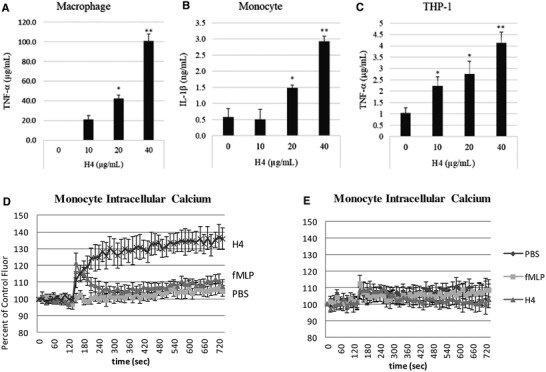
**Histone H4 increases cytokine production and intracellular calcium in human monocytic cells**. (**A**) Human Mϕs, (**B**) human monocytes, and (**C**) THP‐1 cells were treated with indicated concentrations of histone H4 for 24 h. TNF‐α or IL‐1β released in the supernatants was measured by commercially available ELISA kit. Intracellular calcium levels were also elevated in human monocytes treated with histone H4 (**D**). Note again that the histone H4 induced calcium rise is prolonged and not corrected in contrast to that triggered by fMLP. Panel (**E**) repeated the experiments of panel (**D**) but in calcium and magnesium free buffer. Removal of extracellular calcium from the buffer caused a marked drop in the intracellular calcium response to H4 and fMLP (**E**). *N* = 5. Results are presented as mean ± sem (*P ≤ 0.05, **P ≤ 0.01)

To determine if this finding applies to histone H4 activation of other immune cells we tested cytokine production and intracellular calcium responses of monocytic cells. Extracellular histones have been found to increase inflammatory cytokine production in mice.^11^ We here found that histone H4 induced TNF‐α production in human monocyte‐derived macrophages and THP‐1 cells (Figs. 6A and C), and induced IL‐1β (Fig. 6B) production by human monocytes in a concentration dependent manner. Histone H4 caused marked rise in intracellular calcium in monocytes (Fig. 6D), similar to the response seen in neutrophils. Note that removal of extracellular calcium markedly reduced the calcium response to H4 (Fig. 6E). Similar results were obtained with THP cells (not shown).

#### 3.2.2 Histone H4 causes neutrophil membrane depolarization and rapid pore formation in neutrophil membranes

**FIGURE 7:**
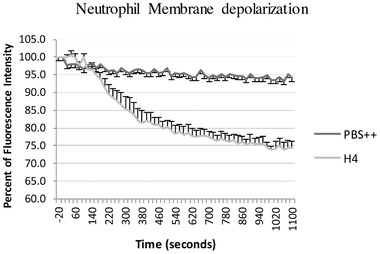
**Histone H4 causes sustained neutrophil membrane depolarization**. Neutrophils were pre‐incubated with 50 mM Di‐OC5 5 min followed by addition of 40 μg/ml of H4 at time zero. Results are mean ± sem for 4 experiments. Neutrophil depolarization was significant (*P* < 0.01) for H4 as compared to cells maintained in PBS alone.

Neutrophil membrane depolarization accompanies respiratory burst activation in response to neutrophil agonists. We tested effects of histone H4 on neutrophil membrane potential and, as in the case of the H4 induced increase in intracellular calcium, H4 caused a sustained depolarization of the neutrophil membrane (Fig. 7).

**FIGURE 8:**
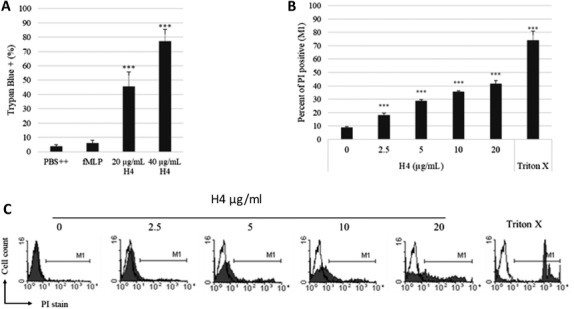
**Histone H4 compromises human neutrophil plasma membranes**. Human neutrophils were incubated with fMLP or histone H4 for 30 min, and cell membrane permeability was assessed by trypan blue dye exclusion (**A**), propidium iodine uptake (**B** and **C**). For trypan blue staining, cells were counted visually after 20 exposure to PBS, fMLP, or H4. Propidium iodide (PI) uptake by human neutrophils treated with PBS control buffer or increasing concentrations of histone H4 for 20 min was measured by flow cytometry. Quantitative analysis of PI positive events is shown in panel (**B**). Representative histograms for PI staining are shown in panel (**C**). Cells treated with control buffer PBS++ are shown in each histogram overlay (black peaks). M1 in each histogram represents the percent of neutrophils that were PI positive. *N* = 5. Results of panels (**A**) and (**B**) are presented as mean ± sem (***P ≤ 0.001)

The findings of sustained elevation of intracellular calcium fed by extracellular sources, and sustained neutrophil membrane depolarization suggested to us that H4 may permeabilize the neutrophil membrane in a way that is not corrected as with other agonists like fMLP. Indeed, after we incubated neutrophils with histone H4 for 20 min, there was a marked increase in trypan blue uptake by the cells. While fMLP only caused 5% of cells to stain blue, 20 μg/ml histone H4 caused 45% of cells to stain blue, and almost 80% of cells was stained blue on 40 μg/ml histone H4 stimulation (Fig. 8A), suggesting that the neutrophil cell membrane was damaged by histone H4. The loss of plasma membrane integrity also led to the uptake of membrane impermeable dye PI that stains intracellular nucleic acids (Fig. 8B). Figure 8C shows representative flow cytometry tracings for PI uptake.

**FIGURE 9:**
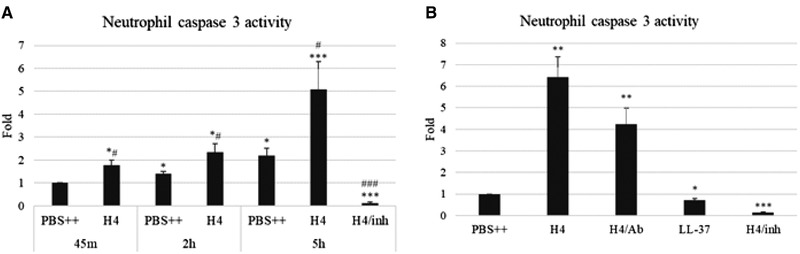
**Effects of Histone H4 human neutrophil caspase 3 activity and LDH release**. (**A**) Neutrophils were treated with 40 μg/ml H4 or PBS++ control buffer for indicated time (45 min, 2 h, or 5 h) and the cells were collected. (**B**) Neutrophils were treated with PBS++ control buffer, 40 μg/ml H4 alone, H4 with anti‐H4 Ab or LL‐37 for 5 h, and the cells were collected. Caspase 3 activity was measured with Z‐DEVD–AMC substrate. H4/inh: Ac‐DEVD‐CHO inhibitor added to H4‐treated samples. The raw units obtained from the assay for PBS‐treated cells at 45 min, 2 h, and 5 h were 158, 202, and 404, respectively. In contrast raw units for H4 treated cells were 296, 519, and 1320, respectively, at these time points. *N* = 5. Results are presented as mean ± sem (significant differences compared with 45 m PBS++ treated cells, *P ≤ 0.05, **P ≤ 0.01, ***P ≤ 0.001; significant differences compared with PBS++ treated cells in matched time groups, ^#^ *P* ≤ 0.05, ^##^ *P* ≤ 0.01, ^###^ *P* ≤ 0.001)

Other assays were performed to assess cell death in response to histone H4. Of interest, despite the membrane permeabilization to trypan blue and PI. Histone H4 increased of neutrophil caspase 3 activity but seemingly over a much slower time course than membrane permeabilization. As shown in Fig. 9A, caspase 3 activity increased over time in control cells, which is consistent with the understanding that neutrophils undergo spontaneous apoptosis. Histone H4‐treated neutrophils displayed higher caspase 3 activity compared to PBS treated control cells at each time point tested. To verify that the increase in caspase 3 activity was due to histone H4 but not other possible contaminants, we added anti‐histone H4 Ab to histone H4‐treated cells and observed a significant decrease in resulting caspase 3 activity induced by histone H4 (Fig. 9B). The specificity of the observed response was confirmed by inclusion of the negative control, Ac‐DEVD‐CHO. LL‐37, another cationic antimicrobial peptide with important immunomodulatory effects, inhibited neutrophil caspase 3 activity (Fig. 9B) as reported elsewhere.^28^, ^29^ LDH release after 20 min incubation with histone H4 was only increased by 9% compared to that seen for control cells in buffer alone or those treated with fMLP (*n* = 5; *P* ≤ 0.001).

#### 3.2.3 Role of signaling cascades and neutrophil receptors in histone H4‐induced respiratory burst responses

**FIGURE 10:**
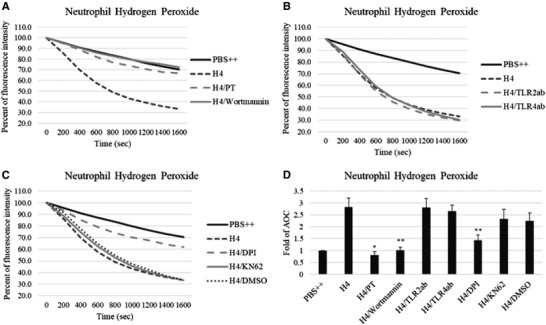
**Effect of metabolic inhibitors and receptor blockers on histone H4‐induced hydrogen peroxide production**. Hydrogen peroxide production in neutrophils on exposure to 40 μg/ml histone H4 for 20 min was measured by assessing the reduction in scopoletin fluorescence (**A–C**). Before adding H4 to neutrophils, cells were untreated or pretreated with 500 ng/ml pertussis toxin (PT) for 2 h or 1 μM wortmannin for 10 min (**A**). Neutrophils were untreated or pretreated with anti‐TLR2 Ab or anti‐TLR4 Ab for 10 min (**B**). Neutrophils were untreated or pretreated with DPI or KN62 for an hour (**C**). (**D**) Fold changes of area over the curve (AOC) from (**A**) to (**C**). *N* = 5. Results are presented as mean ± sem (*P ≤ 0.05, **P ≤ 0.01, ***P ≤ 0.001)

**FIGURE 11:**
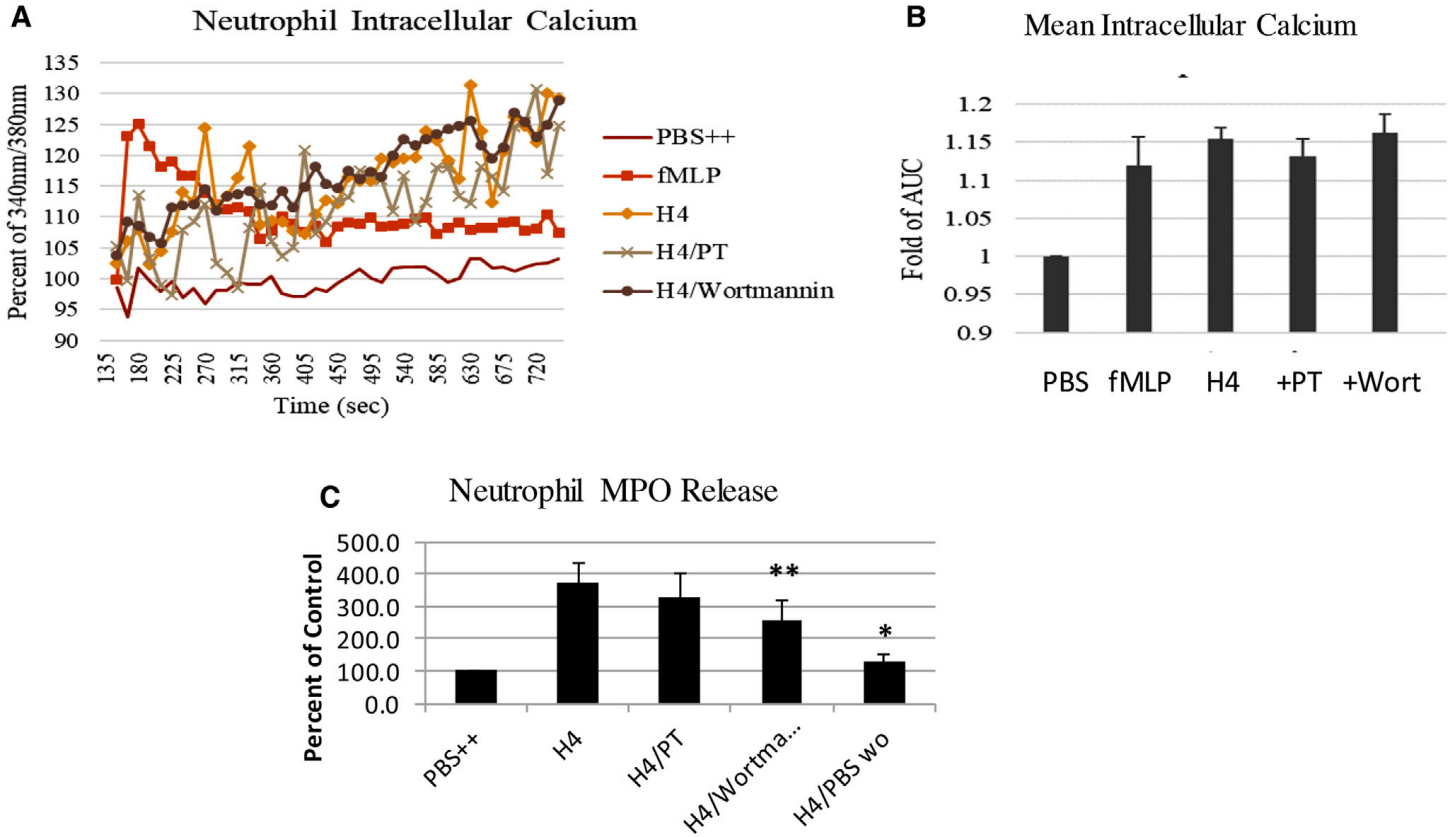
**Effects of PT and wortmannin on intracellular calcium response and MPO release to histone H4**. Intracellular free calcium changes in neutrophils on exposure to 40 μg/mL histone H4 for 20 min were measured by detecting the fluorescence of Fura‐2 AM loaded cells. Before adding H4 to neutrophils, cells were untreated or pretreated with 500 ng/ml pertussis toxin (PT) for 1 h or 1 μM wortmannin for 10 min (**A**). (**B**) Fold changes of area under the curve (AUC) from (**A**). H4 again caused significant elevation of intracellular calcium but the inhibitors did not reduce this rise. In panel (**C**), neutrophil MPO release was measured in PBS with calcium and magnesium (H4), or in cells treated with PT or wortmannin (H4/PT or H4/wortman). In addition, MPO release to H4 was measured in PBS without added calcium and magnesium (H4/PBSwo). *N* = 5. Results are presented as mean ± sem (*P ≤ 0.05, **P ≤ 0.01)

To determine which signaling cascades might contribute to histone H4 induced neutrophil activation, we pre‐incubated neutrophils with pertussis toxin (a blocker of G protein signaling) and wortmannin (a PI3K inhibitor). As shown in Figs. 10A and D, both of these treatments significantly reduced H4 stimulated H_2_O_2_ production. As noted in the introduction several papers have implicated binding of histones to TLRs as the cause of activation of various cells, including macrophages. As shown in Figs. 10B and D, blocking Abs directed against TLR2 and 4 did not alter H4 stimulated H_2_O_2_ production. Similarly blocking the ionotropic P2X_7_ receptor (P2X_7_R) that binds extracellular ATP with the P2X_7_R antagonist, KN62, did not block these responses (Fig. 10C). As a control neutrophils were pretreated with diphenyleneiodonium (DPI) and this did block the H4 stimulated H_2_O_2_ production. Again mean ± sem areas under curve for Figs. 10A‐C are shown in Fig. 10D. We tested whether PT or wortmannin would alter the intracellular calcium rise caused by histone H4. As shown in Figs. 11A and B, there was no significant effect of either PT or wortmannin on histone‐induced calcium response. There was a significant reduction in H4‐induced MPO release in presence of wortmannin and a more marked reduction in MPO release when the assay was carried out in calcium and magnesium free buffer with added EGTA (Fig. 11C).

## 4 DISCUSSION

Prior reports have shown that cell‐free histones trigger potent inflammatory responses and may be involved in the pathogenesis of sepsis and injury to various tissues, including the lung. There have been limited studies regarding direct effects of histones on neutrophils. One study showed that extracellular histones induced NETs formation but were not able to induce ROS production from neutrophils.^6^ Another study found that histones can induce NET formation from mouse neutrophils in vitro.^4^ We now demonstrate that histone H4 is a profound stimulus of human neutrophil activation with widespread effects on neutrophil respiratory burst, adhesion, cytokine release, and degranulation. We suspect a similar mechanism for cytokine generation by monocyte/Mϕs in response to histone H4 that we demonstrated, although further research will need to be done to confirm that. The respiratory burst and degranulation responses are shown to be dependent on elevation of intracellular calcium mainly from extracellular sources. In turn the influx of calcium is the result of permeabilization of the neutrophil membrane by histone H4. This permeabilization was demonstrated by several assays, including membrane depolarization and entry of trypan blue and PI into the cells. These findings are in agreement with other recent studies that showed formation of membrane pores in airway smooth muscle cells and permeabilization of endothelial cells causing calcium influx by histone H4.^21^, ^23^ We believe that this membrane permeabilization is possibly a key mechanism through which histones or at least histone H4 mediates severe inflammatory responses and should be evaluated for other key cell types such as monocytes and platelets. Note that we found similar calcium influx caused by H4 in human monocytes.

Based on blocking studies with Abs and inhibitors, we could not show evidence that TLR2, TLR4, or ATP receptors are involved in neutrophil ROS production induced by histone H4. We cannot exclude that histones may bind to other receptors on neutrophils. One important caveat is that the later effects seen (e.g., MPO and IL‐8 release) could be secondary effects of cell lysis. A prior report showed that respiratory epithelial cells released large amounts of IL‐8 and Mϕ inhibitory factors despite undergoing necrosis in response to influenza virus infection.^30^ Our finding that extracellular calcium chelation blocked MPO release suggests either that removal of calcium reduces cell lysis caused by histone H4 or inhibited a signaling cascade leading to degranulation. In any case, release of MPO and IL‐8 through any mechanism would be pro‐inflammatory in vivo.

In initial experiments, we found that the other arginine rich histone, histone H3, had similar neutrophil activating effects, whereas histones H2a and H2b and calf thymus histones did not. This suggests that neutrophil activation is a property of the arginine rich histones. Of interest, we also found that the arginine rich histones, H3 and H4, had influenza neutralizing activity, whereas the lysine rich histones, H2A and H2B, did not.^3^ We here show that a complex of H3 with H4 did not cause neutrophil ROS production, suggesting that histones H3 and H4 lose the property of activating neutrophils when bound to each other. These initial studies must be taken with a grain of salt since the other histone preparations (apart from calf thymus histones) contained endotoxin. For the rest of our studies, we focused on histone H4 alone which in any case has been importantly implicated in stimulating inflammatory responses and being elevated during inflammatory injury in vivo in other studies.^6^, ^21^, ^23^

Histone H4 appears to induce a form of cell death, although surprisingly it was not gross cell lysis at the early stage since LDH release was only modestly increased at 20 min despite extensive uptake of trypan blue and PI. This suggests that the membrane pores induced by histone H4 at least at early time points allow molecules of trypan blue or PI (872 and 668 g/mol) rapid entry but are less permeable to larger molecules like LDH (35735 g/mol). There does not appear to be an active closing of the pores since at least over 20 min calcium continues to enter the cells and the membrane remains depolarized. With further time caspase 3 is activated in the cells. Further studies of histone H4 induced cell death would be of interest since it has also been shown to cause inflammasome activation in Kupffer cells^7^ and we show that it can stimulate IL‐1 production without additional stimuli in monocytes. A recent paper showed that influenza virus induces a mixed form of cell death with features of apoptosis, pyroptosis, and necrosis termed panoptosis through activation of caspase 6.^31^ Further studies of histone induced inflammation or NET induced inflammation in vivo could evaluate the role of caspase 6, since histones and NETs are implicated in pathophysiology of influenza.^4^ In any case, we cannot definitely categorize the ultimate fate of neutrophils treated with histone H4 over the longer term, although it has characteristics of both apoptosis and necrosis based on our assays.

Histones are the main protein components of neutrophil extracellular traps (NETs), and histones can also be released from dead cells. It is still unclear what are the sources of cell‐free histones or H4 measured in lung samples or serum in vivo during inflammation. It will be important to identify the source and physiological concentrations of circulating histones in different kinds of infectious or inflammatory conditions. Abrams et al. investigated 250 patients with severe trauma and found that circulating histone levels ranged from 10 to 230 μg/ml within 4 h after injury, and the level peaked at 24 h and remained detectable after 72 h.^6^ In our study, we used histone H4 up to 40 μg/ml, which is within this range.

Of interest, although we propose that membrane permeabilization and the resultant calcium influx is the main trigger for neutrophil activation caused by histone H4 (and possibly H3), we did find that the signal transduction inhibitors pertussis toxin (PT) and wortmannin were able to ablate H_2_O_2_ production while not inhibiting the calcium rise caused by histone H4. In parallel experiments, these inhibitors did not alter the intracellular calcium rise caused by histone H4. It is possible that the observed blocking effects of PT and wortmannin reflect triggering of these signaling cascades not through receptor mediated events but through secondary activation events possibly initiated by the calcium rise within the cell. Obviously this is somewhat speculative but consistent with our data.

As noted in the introduction, cell‐free histones and/or NETs have been implicated in a range of severe inflammatory conditions. Our findings suggest that one aspect of this inflammatory cascade is activation of neutrophils by histone H4. This could lead to a vicious cycle in which activated neutrophils recruit more neutrophils and trigger additional activation of other immune cells like monocyte/macrophages. Of interest, in addition to a damaging role in severe influenza virus infection,^4^ NETs have been implicated now as a trigger for the profound inflammation and hyper‐coagulability seen in severe COVID‐19.^17^, ^18^ Given these effects of cell free histones, it is not surprising that various mechanisms appear to have evolved to bind or scavenge histones in the body. Several histone scavengers have been reported or proposed, including C‐reactive protein (CRP), activated protein C (APC), heparin, albumin, recombinant thrombomodulin (rTM), and pentraxin 3 (PTX3).^1^, ^11^, ^21^, ^32^, ^33^, ^34^ In some models, anti‐histone Abs have been shown to be protective versus lethal effects of histones.^4^, ^11^ Methods to inhibit NET and histone release or blunt the effects of histones in vivo should be a high priority for future studies.

## AUTHORSHIP

I‐Ni Hseih designed and did most of the experiments and wrote the original manuscript draft. Xavier Deluna carried out some experiments as did Mitchell White. Kevan Hartshorn oversaw the project, helped design experiments and revised the manuscript.

## DISCLOSURE

The authors declare no conflict of interest.
